# Chitosan-Based Nanofibrous Membrane Unit with Gradient Compositional and Structural Features for Mimicking Calcified Layer in Osteochondral Matrix

**DOI:** 10.3390/ijms19082330

**Published:** 2018-08-08

**Authors:** Jiaoyan Liu, Qing Fang, Xiaofeng Yu, Ying Wan, Bo Xiao

**Affiliations:** 1 College of Life Science and Technology, Huazhong University of Science and Technology, Wuhan 430074, China; liujiaoyan@hust.edu.cn (J.L.); m201671610@hust.edu.cn (Q.F.); m201771729@hust.edu.cn (X.Y.); 2 Institute for Clean Energy and Advanced Materials, Faculty for Materials and Energy, Southwest University, Chongqing 400715, China

**Keywords:** chitosan, silk fibroin, hydroxyapatite, nanofibrous membrane unit, layered scaffold, calcified layer

## Abstract

Chitosan (CH), silk fibroin (SF), and hydroxyapatite (HA) were used to prepare CH/SF/HA composites and the resulting composites were electrospun into nanofibrous membrane units with gradient compositional and structural features. The optimal membrane unit was used together with CH/HA and CH/SF composites to fabricate a type of three-layer scaffold that is intended for osteochondral repair. The bottom layer of the scaffold was built with CH/HA composites and it served as a subchondral layer, the integrated nanofibrous membrane unit functioned as the middle layer for mimicking the calcified layer and the top layer was constructed using CH/SF composites for acting as a chondral layer. The nanofibrous membrane unit was found to be permeable to some molecules with limited molecular weight and was able to prevent the seeded cells from migrating cross the unit, functioning approximately like the calcified layer in the osteochondral matrix. Layered scaffolds showed abilities to promote the growth of both chondrocytes and osteoblasts that were seeded in their chondral layer and bony layer, respectively, and they were also able to support the phenotype preservation of seeded chondrocytes and the mineralization of neotissue in the bony layer. Results suggest that this type of layered scaffolds can function as an analogue of the osteochondral matrix and it has potential in osteochondral repair.

## 1. Introduction

Tissue engineering, which usually involves in the combined use of porous scaffolds, seed cells, and pertinent signal molecules, has emerged as a new technique for restoring the impaired tissues in the past decade. In general, the employed scaffolds need to be biocompatible, biodegradable, and easy to be processed into the desired shapes without inducing unwanted effects, such as inflammation, allergenic reactions, toxicity, carcinogenesis, and so on [1,2,3]. To date, a variety of materials and techniques have been used for fabricating different types of scaffolds that are propitious to tissue repair [2,3,4]. When considering the fact that scaffolds serve as temporary extracellular matrix (ECM) and can exert complex impacts on the growth, differentiation, migration, distribution, and orientation of the seeded cells, much attention has been paid to the compositions, structures, and properties of scaffolds in order to achieve better results in tissue repair. It is known that most human tissues have inhomogeneous compositions and anisotropic structures, highly relying on the varieties of cells and ECM, and therefore, the scaffolds having inhomogeneous compositions, anisotropic structures, and biomimetic features have been rapidly developed in recent years, since the scaffolds with homogeneous compositions and isotropic structures can only fulfill limited needs in tissue repair [2,3,4,5,6]. Among anisotropic scaffolds, stratiform scaffolds with heterogeneous compositions have attracted a lot of attention in the field of osteochondral repair because they are able to partially mimic the inhomogeneous compositions, stromatolithic structures, and layer-dependent properties of osteochondral tissues [7,8,9].

Articular cartilage is a specific type of tissue that imparts joints with several critical functions [10]. Anatomically, articular cartilage consists of spatially identifiable four layers, which are commonly named as superficial layer, intermediate layer, deep layer, and calcified layer [9,11]. These layers constitute a complex organization that has layer-dependent alternations in compositions, structures, and properties, and moreover, these alternations are quite irregular in direction [5,8,9,10]. Among these layers, the calcified layer acts as a special transition zone that tightly connects the hyaline cartilage and the subchondral bone, and meanwhile, functions as a vital physical barrier to prevent vascular invasion and the calcification of hyaline cartilage [9,10,11]. In addition, the calcified layer has a semi-permeable characteristic and it allows for some molecules with limited sizes to pass through to maintain a stable microenvironment for the survival of chondrocytes [10,11]. Bearing in mind the specificity and importance of the calcified layer, a rational strategy in fabrication of stratified scaffolds that are used for osteochondral repair is to endow the scaffolds with a mimetic structure containing a chondral zone, a calcified layer and a subchondral zone as similar as possible to promote the integral reconstruction of osteochondral tissues.

Several efforts have been made to fabricate layered scaffolds, including bi-layered, tri-layered, and even multilayered scaffolds, by using different materials and processing techniques, and these layered scaffolds can partially mimic certain characteristics and properties of osteochondral ECM [11,12,13,14,15,16,17,18]. The major organic materials that are used for building layered scaffolds include collagen, silk fibroin, agarose, hyaluronic acid, and cell-free ECM, as well as some biodegradable synthetic polyester [12,13,14,15,16,17,18,19,20]. In the cases of scaffolds that are used for osteochondral repair, inorganic materials, such as hydroxyapatite, tricalcium phosphate, and bioactive glass are also employed because the calcified layer and subchondral layer of native osteochondral ECM contain various amounts of inorganic matter besides other ingredients, such as proteins and glycosaminoglycans (GAGs) [13,14,16,21,22,23]. To date, despite the growing variety of the layered scaffolds, the ingeniously constructed stratiform scaffold that contains well-controlled biomimetic calcified layer, and can facilitate the regeneration of osteochondral tissues with stable and durable function maintenance is still few [10,21,24,25]. 

In this study, an effort was made to fabricate a new type of layered scaffold in which a prebuilt nanofibrous membranous unit with gradient compositional and structural features was integrated to mimic calcified layer, and two other layers were constructed to function as a chondral layer and a subchondral layer. The nanofibrous membranous unit was prepared by electrospinning chitosan/silk fibroin/hydroxyapatite composites, and the chondral layer and the subchondral layer were fabricated with chitosan/silk fibroin and chitosan/hydroxyapatite composites, respectively. These scaffolds were characterized for their basic structures and major properties. In addition, chondrocytes and osteoblasts were seeded onto the layered scaffolds and co-cultured in vitro to evaluate the suitability of scaffolds for potential applications in osteochondral repair.

## 2. Results and Discussion

### 2.1. Nanofibrous Membranes Units

Figure S1 shows a representative TEM image of presently synthesized hydroxyapatite (HA) nanoparticles (NPs), and their X-ray diffraction (XRD) pattern. The TEM image denotes that HA NPs were in the shape of needle or shuttle, and their sizes along their length changed from several tens of nanometers to around 150 nanometers. The XRD pattern for these HA NPs exhibits that a few strong diffraction peaks were located at around 26°, 32° (the synaptonemal complex of three peaks), and 39°, and they are typically indicative of the summed contribution of the lattice planes of HA NPs [26]. 

Chitosan (CH) has many good properties and its chemical structure is similar to that of GAGs that largely exist in native ECM of different body tissues, thus making CH a popular biomaterial for a wide variety of applications in tissue engineering, drug delivery, and gene vectors [27,28]. Despite its merits, CH scaffolds usually have poor wet-state strength and are degraded fast in vivo, which hinder their applicability to situations where the high strength and degradation tolerance are needed. Silk fibroin (SF) is a fibrous protein with demonstrated advantages, and SF scaffolds show robust wet-state strength and have slower in vivo degradation rate as compared to that made from some other natural polymers, such as collagen, starch, and CH [28,29,30]. In this study, CH was used together with SF for preparing nanofibers or composites in order to endow them with enhanced wet-state strength and improved degradation tolerance. Several studies mentioned that poly (ethylene oxide) (PEO) was a suitable additive for CH/HA-related nanofibers, because it can facilitate the dispersion of HA NPs while smoothing the surface of fibers [29,31,32]. Accordingly, a small amount of PEO was used for the present nanofiber preparation. To achieve some nanofibrous membranes with gradient compositional features, CH, SF, and HA in membranes were formulated in such a way that each of them had their own compositional gradient when different membranes were compared each other, as illustrated in Table 1. Besides the effects of component proportions, processing parameters can also significantly affect the morphology and the property of nanofibrous membranes [33]. Several major processing parameters, including dope concentration, flow rate, tip-collector distance, and working voltage, were thus optimized to yield bead-free, continuous, and uniform nanofibers in the membranes.

**Figure 1:**
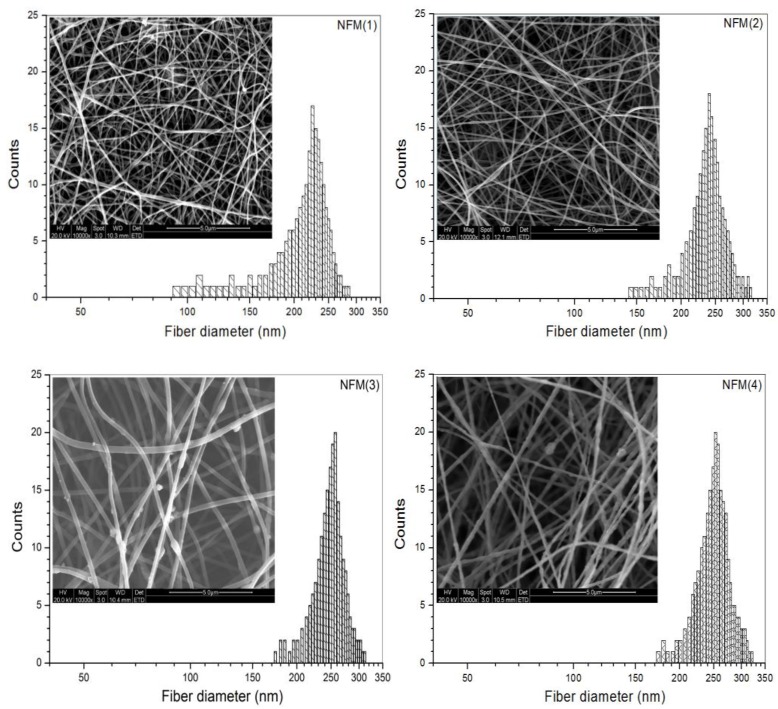
Micrographs of nanofibrous membranes and diameter distributions of nanofibers (see Table 1 for their parameters).

Figure 1 presents several morphologic micrographs for four kinds of nanofibrous membranes (NFM(*i*), *i* = 1, 2, 3, 4) and the diameter distributions of nanofibers in the membranes. Fibers in the NFM(1) membrane seemed to be somewhat stuck, but it had smooth surface, and their diameter changed in a range between about 90 and 290 nm. In the case of the NFM(2) membrane, fibers were dispersedly stacked without sticking and their diameter-distribution interval became narrower when compared to that shown in NFM(1). With respect to NFM(3) and NFM(4) membranes, many small granulates were seen to be protruded from fibers, which can be ascribed to the HA aggregates that were formed during electrospinning due to the higher HA content in HA-trapped nanofibers [30]. Fibers in NFM(4) membrane were formulated with a feed HA amount of 31 wt% (see Table 1), and the image for NFM(4) membrane reveals that fibers in the membrane had very rough surface due to the presence of protruded granulates, signifying that a high HA content in fibers is unfavorable for the smooth and uniform formation of fibers. Based on comparative trials, it was found that a further increase in HA content in fibers could frequently lead to the discontinuity of fibers, and therefore, the feed HA amount was controlled at around 30 wt% or less in order to achieve continuous CH/SF/HA nanofibers.

Two processing parameters, spinning time and tip-collector distance, were simultaneously regulated using orthogonal design to endow NFM(*i*) (*i* = 1, 2, 3, 4) membranes with similar thickness but different average pore-sizes and porosities, and relevant results are also provided in Table 1. Data numerated in Table 1 indicate that, by doing so, the pore-size and porosity of these membrane increased in similar trends when viewing from NFM(1) to NFM(4), and significant differences in both the average pore-size and average porosity were detected among them. Based on these data, it can be envisioned that the entirety assembled by NFM(*i*) (*i* = 1, 2, 3, 4) membranes would have different gradients in the composition, pore-size, and porosity if these membranes were piled up in a designated order changing from NFM(1) to NFM(4).

**Figure 2:**
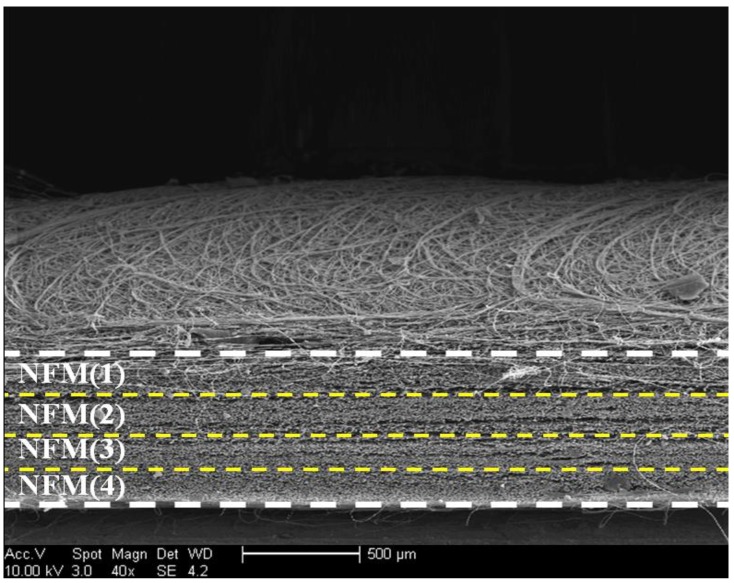
Scanning electron microscope (SEM) image for cross-section of membrane unit assembled by unremittingly spinning NFM(*i*) (*i* = 1, 2, 3, 4) nanofibrous membranes together.

In principle, it seems to be feasible to superimpose NFM(*i*) (*i* = 1, 2, 3, 4) membranes together to form an assemblage, and the resultant assemblage could be further integrate into a layered scaffold. Nevertheless, this approach was found to be impractical because the adjacent nanofibrous membranes cannot be tightly bound together via simple superposition, which could result in a disconnection between neighboring membranes during the post scaffold processing. A nanofibrous membrane unit was thus prebuilt by spinning four kinds of nanofibrous membranes together in a layer-by-layer manner in a designated order of NFM(4), NFM(3), NFM(2), and NFM(1). Since the nanofibrous membrane unit was prepared by unremittingly spinning, the posteriorly spun nanofibrous membrane are able to bind to the previously spun membrane that is not yet fully dried during the electrospinning, leading to the formation of well bound nanofibrous membrane units. A representative SEM image for the cross-section of the resulting nanofibrous membrane unit is displayed in Figure 2. It can be seen that many membranous layers were well overlaid each other in parallel to form a compact nanofibrous membrane unit with a thickness of around 600 μm. Based on the parameters that are listed in Table 1, it can be drawn that such prepared nanofibrous membrane unit has gradient compositional features while being endowing with gradient porous structures. So, a prebuilt nanofibrous membrane unit will be integrated into the layered scaffolds for mimicking calcified layer.

### 2.2. Fabrication of Layered Scaffolds

**Figure 3:**
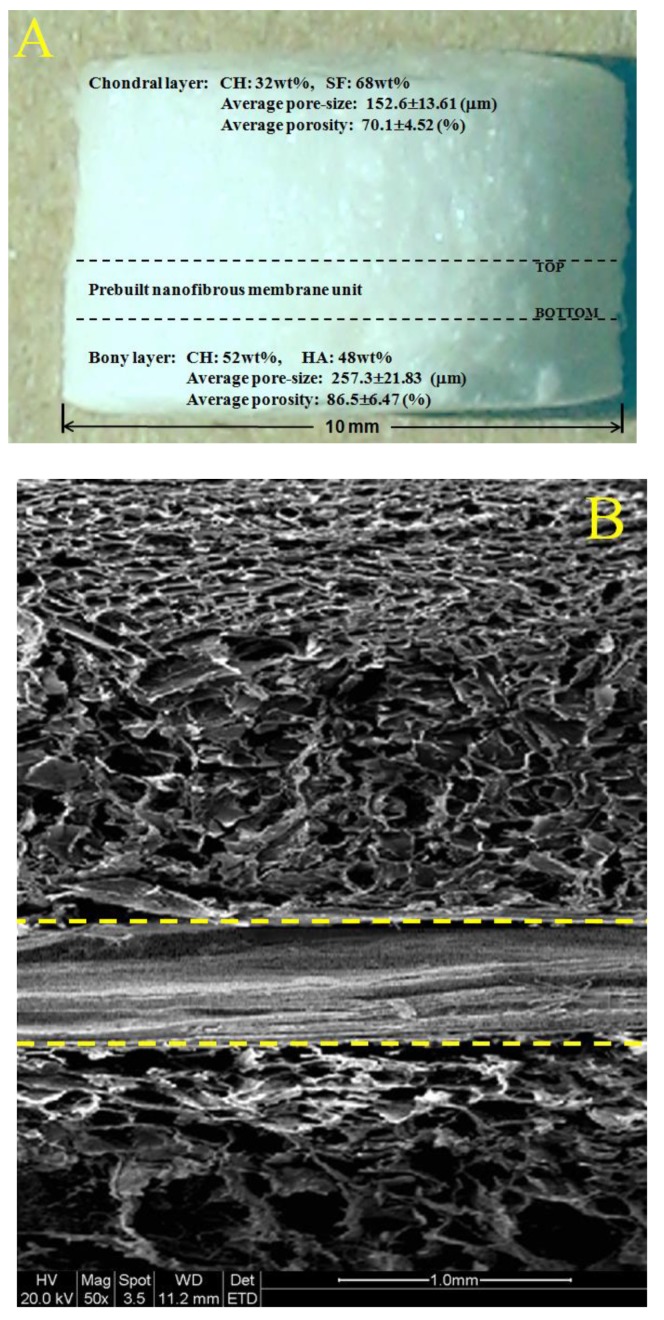

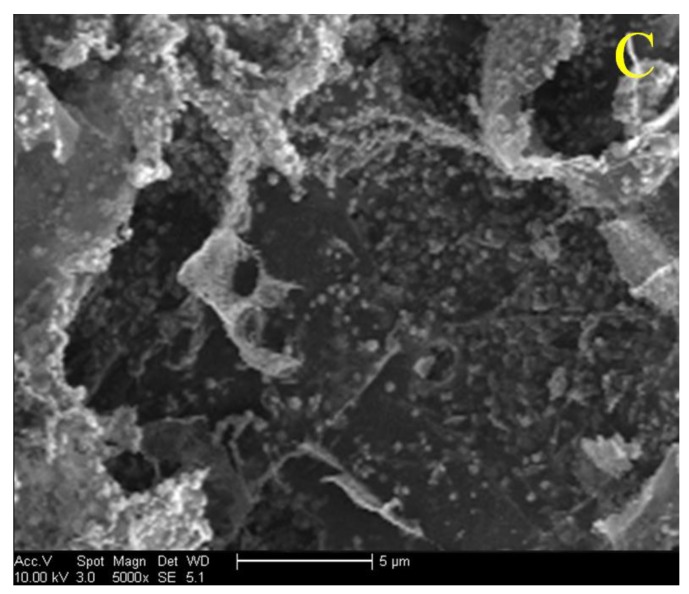
Photo of layered scaffold with designated compositions (**A**) the thickness for chondral layer, mimetic calcified layer and bony layer is about 1.8 mm, 0.6mm and 1.2 mm, respectively; SEM image taken from the vertical section of layered scaffold (**B**) yellow dashed lines in the image indicate interface between different layers; and, SEM image with high magnification for the site located in bony layer (**C**) of layered scaffold.

In the field of osteochondral repair, one of major challenges is to develop suitable scaffolds that have distinct but integrated layers and that are able to effectively mimic osteochondral ECM [6,7,8]. In this study, we intend to build layered scaffolds that have gradient compositions and certain gradient structural features. Figure 3A shows a photo of layered scaffold with designated composition proportions for chondral layer and bony layer. The bony layer of the scaffold is designed to serve as a subchondral bone layer, and this layer was built while using a CH/HA composite to endow the layer with high strength, large pore size, and high porosity. The prebuilt nanofibrous membrane unit was overlaid the bony layer by placing the bottom of the unit (see Figure 2 and Figure 3) against the top of the bony layer, and this new layer is functioned as a mimetic calcified layer. Atop the mimetic calcified layer, a chondral layer was constructed using a CH/SF composite.

As shown in Figure 2, the top layer of the nanofibrous membrane unit contains a less amount of CH or HA, but a higher amount SF (see Table 1), and meanwhile, has smaller average pore-size and lower porosity when compared with other layers in the nanofibrous membrane unit. It is known that the osteochondral ECM mainly consists of proteins, GAGs, proteoglycans, glycoproteins, and inorganic ingredients [5,6,8]. By overlaying the bottom of the nanofibrous membrane unit with the bony layer and connecting the top of the nanofibrous membrane unit with the chondral layer, CH, and SF components, each changes in a gradient manner from the chondral layer to the bony layer in the opposite direction (see Figure 2 and Figure 3A and Table 1), are able to mimic the gradient compositions of GAGs and major proteins in the osteochondral ECM, respectively. Further, the HA content in the layered scaffold varied from the nanofibrous membrane unit to the bony layer in an increasing trend, and it is approximately consistent with the alteration of major inorganic components that are distributed in the calcified layer and the subchondral layer in the osteochondral ECM. Therefore, the presently developed nanofibrous membrane unit is able to function as a mimetic calcified layer with certain compositional and structural similarities to the native calcified layer in the osteochondral ECM. 

Figure 3B presents a representative SEM image taken from the vertical section of layered scaffold. It can be seen that (1) there were many pores distributed in the chondral layer, and many large pores were viewed in the bony layer; (2) the porosity of the bony layer was estimated to be much higher than that for the chondral layer; and, (3) the chondral layer and the bony layer were well connected by the nanofibrous membrane unit without the appearance of crannies at two interfaces. This image verifies that the presently constructed nanofibrous membrane unit can function similarly to the native calcified layer in the osteochondral ECM in which the hyaline cartilage and the subchondral bone are tightly connected by the calcified layer.

A SEM image that was taken from a site located in the bony layer with high magnification is represented Figure 3C. This image shows that many small granulates with various sizes were seen to be embedded in the matrix. These granulates can be ascribed to HA NPs or their aggregates. The size of granulates in Figure 3C is estimated to be in a range between 400 and 800 nm, several times larger than that of HA NPs shown in Figure S1, confirming that HA NPs have been incorporated into the CH/HA composite, and some of them have undergone various degrees of aggregation during the preparation of CH/HA composites due to the physical mixing of the two components.

### 2.3. Permeable Properties of Mimetic Calcified Layer

**Figure 4:**
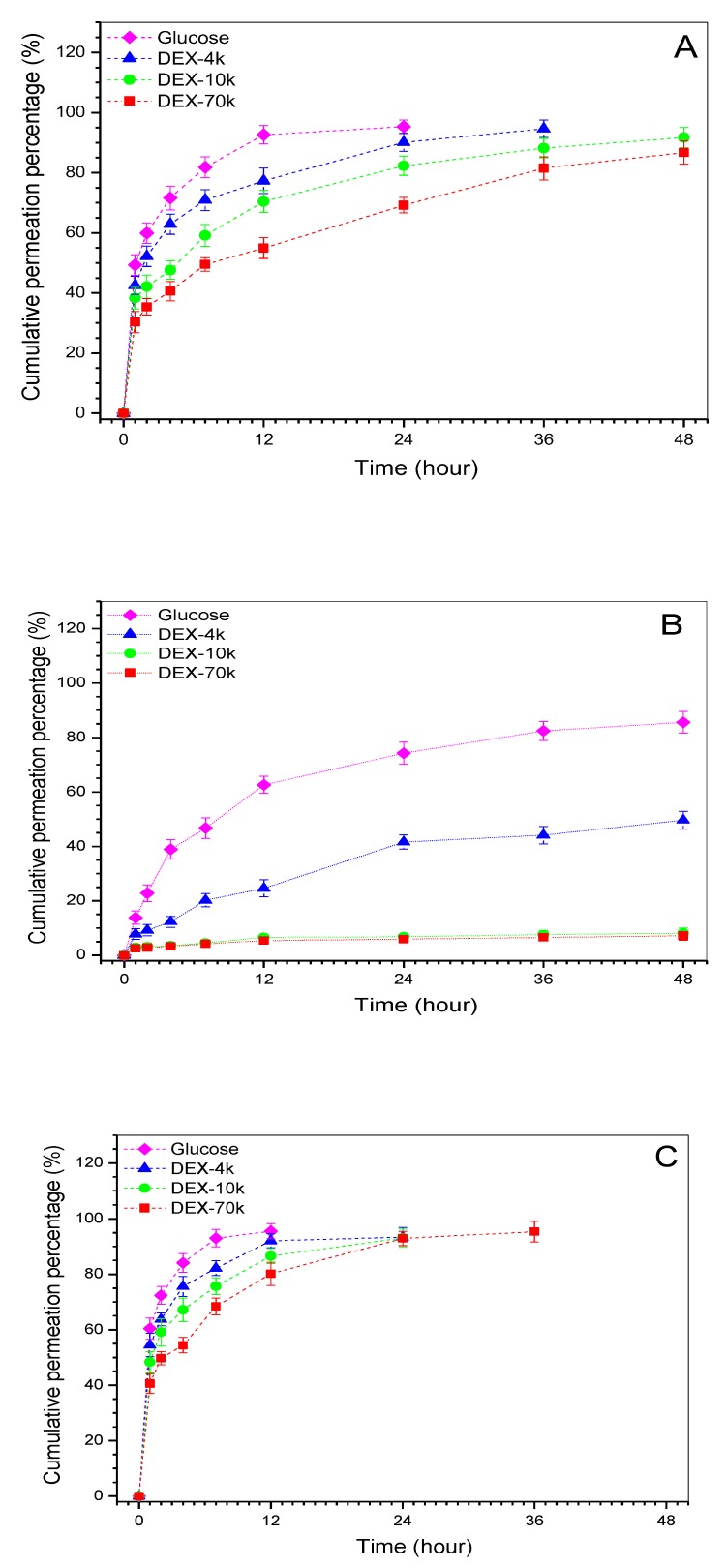
Time-dependent sheet permeation of different model molecules correspond to chondral layer (**A**); mimetic calcified layer (**B**); and, bony layer (**C**), respectively, (see Figure 3A for the name of layers).

Several model molecules were used to test the permeability of different layers and the obtained data are represented in Figure 4. It can be observed that the chondral layer and the bony layer were permeable to the tested molecules but these model molecules permeated cross the layers at varied rates. In the case of the mimetic calcified layer, about 84% glucose was able to diffuse through the layer after 48 h permeation, and the matched permeability for DEX-4k reached ca. 48%. In regard to DEX-10k and DEX-70k, their permeability was around 6% or less within the same sampling time interval, revealing that this layer is only permeable to certain molecules with limited molecular weight. There were significant differences (*p* < 0.05) in the permeability among three layers when the same model molecule was compared. In addition, the mimetic calcified layer showed an affirmatory ability to block the passage of the molecules with molecular weight higher than 10 k.

When considering that the solutions that were prepared by these model molecules had the same concentration, the hydrated size of the model molecules should thus be proportional to their respective molecular weight [34]. Significant differences in permeation rates between the chondral layer and the bony layer can be ascribed to their different average pore sizes and porosities. The layer with larger average pore size and higher porosity would certainly facilitate bigger model molecules to permeate through than that having smaller average pore size and lower average porosity. In particular, curves for the mimetic calcified layer in Figure 4B confirm that the nanofibrous membrane unit has a semi-permeable feature and it is able to serve a very similar role in transportation of low molecular weight substances between the chondral layer and the bony layer when compared to the native calcified layer in the osteochondral ECM [10,11,16].

### 2.4. Cell Culture

**Figure 5:**
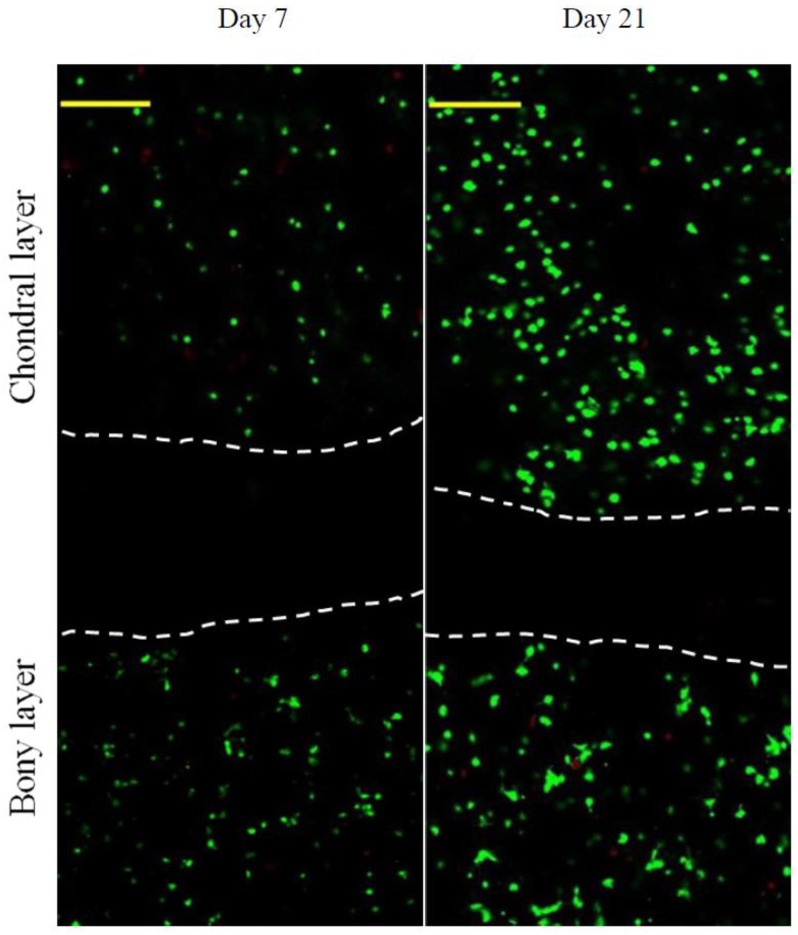
Confocal microimages for live/dead-staining of cells (vertical sections of the layered scaffold seeded with chondrocytes in chondral layer and osteoblasts in bony layer). Images show z-projection of a 100 μm z-stack (green, viable cells; red, dead cells; scale bar, 250 μm; areas between white dashed lines indicate the cell-free zones and correspond to the mimetic calcified layer).

The chondral layer and the bony layer of scaffolds were seeded with chondrocytes and osteoblasts, respectively, and the cell-seeded scaffolds were co-cultured in two-compartment chambers (see Figure S2) to assess the effects of scaffolds on the growth of chondrocytes and osteoblasts. Two representative images for the stained cells after incubation for various durations are presented in Figure 5. After seven-day culture, very few dead cells were imaged in the chondral and the bony layers, meaning that both chondrocytes and osteoblasts had high viability. After 21-day culture, cell density in both chondral and bony layers increased significantly and most of the cells had well-maintained viability since there were few dead cells viewed in two layers. In particular, chondrocytes and osteoblasts were separated by a cell-free zone during the culture periods, demonstrating that the integrated nanofibrous membrane unit inside the layered scaffold is able to function like a mimetic calcified layer in the osteochondral ECM for maintaining the survival of both chondrocytes and osteoblasts in their own regions.

Cell proliferation in chondral and bony layers was assessed by measuring the cell number, and data are graphed in Figure S3. Bar-graphs in Figure S3A explicate that the growth of chondrocytes roughly experienced two phases: fewer cells grown from day 1 to day 3; and, after that, cells grown relatively fast, with significant differences being detected between adjacent sampling time points. A similar cell growth trend was also recorded for osteoblasts in the bony layer (see Figure S3B). When considering the initial number of the seeded cells, results in Figure S3 suggest that the layered scaffolds are able to well support the growth of both chondrocytes and osteoblasts in their respective layers.

### 2.5. Matrix Deposition Assessment

**Figure 6:**
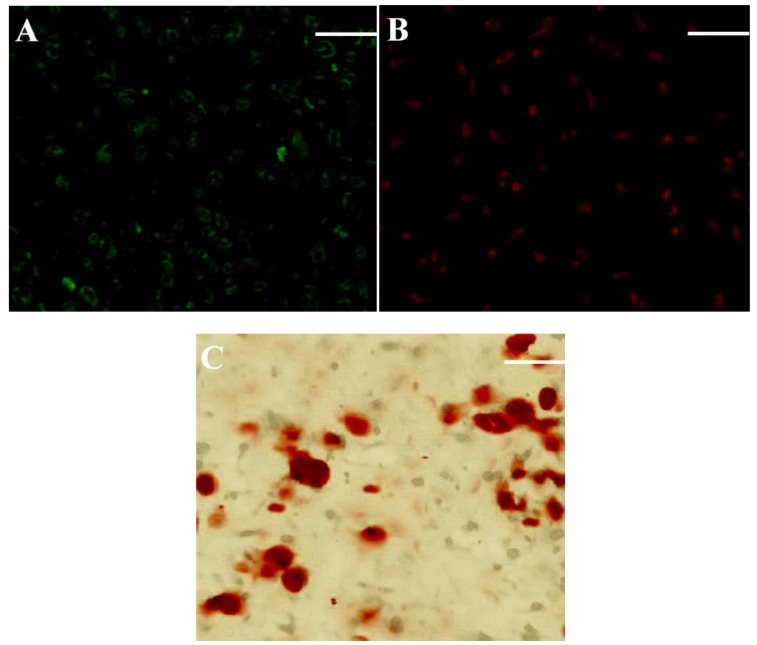
Typical staining micrographs for sections vertically cut from chondral layer (**A**) and bony layer (**B**,**C**) of cell-seeded scaffold (**A**, Immunofluorescence staining for type-II collagen; **B**, Immunofluorescence staining for type-I collagen; and **C**, Alizarin red staining for calcium deposition; culture time: three weeks; Scale bar, 100 μm).

**Figure 7:**
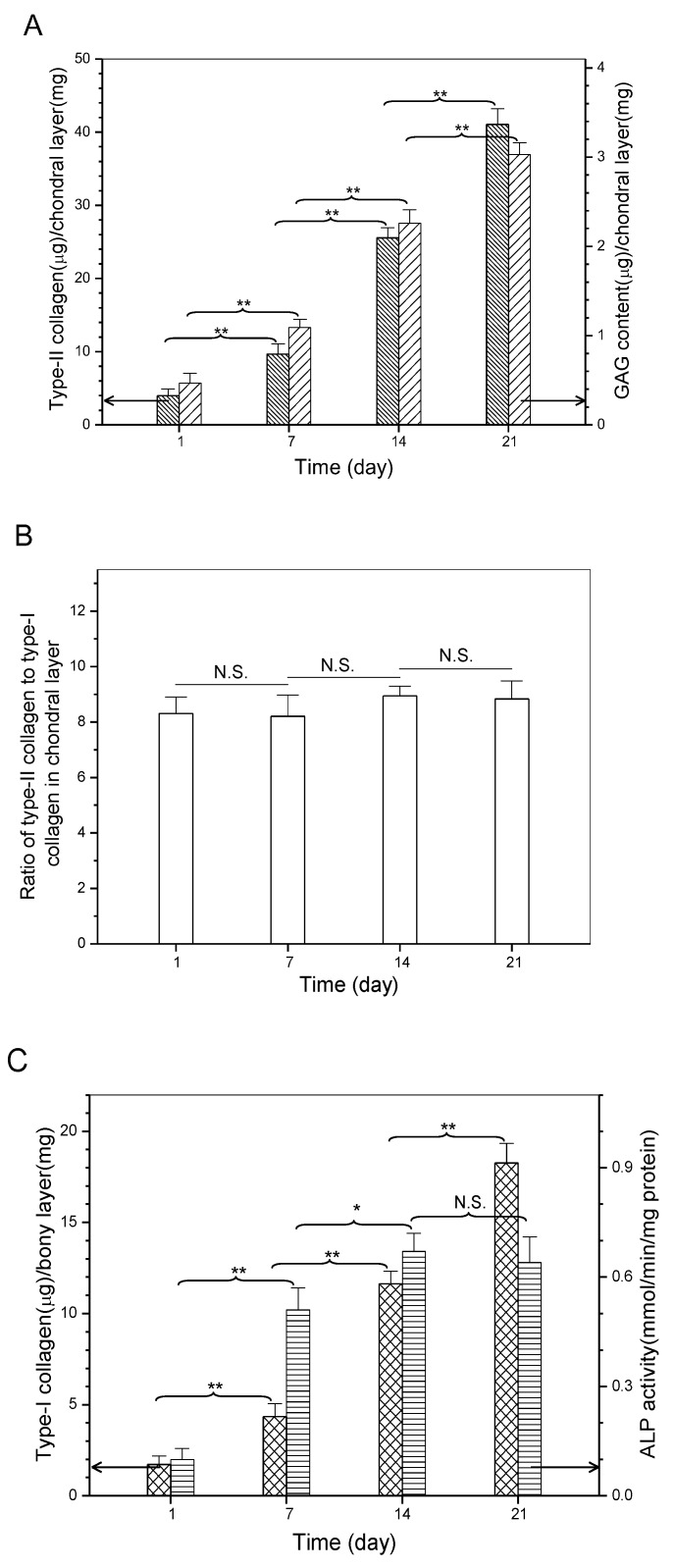
Biochemical assessment of matrix molecules in chondral layer (**A**,**B**) and bony layer (**C**) of layered scaffolds (* *p* < 0.05; ** *p* < 0.01; N.S., no significance).

It is known that chondrogenesis is associated with several marker molecules, typically including type-II collagen, GAG, and aggrecan, which help in constructing the cartilaginous ECM [10,11,35]. On the other hand, type-I collagen secreted by osteoblasts and the followed formation of calcium nucleates in newly synthesized ECM act as two of important indicators for the progression of osteogenesis [10,21,36]. Sections, respectively, obtained from the chondral layer and the bony layer of cell-seeded scaffolds were stained and representative micrographs are represented in Figure 6. Immunofluorescence staining was evident in the deposition of type-II (Figure 6A) and type-I (Figure 6B) collagens, and calcium (Figure 6C) was already deposited in the bony layer of layered scaffolds, respectively. Quantitative analysis of type-I, type-II collagens, and GAGs was conducted, and relevant data are depicted in Figure 7. The production of type-II collagen and GAGs rapidly increased over the culture time (Figure 7A). In particular, ratio of type-II collagen to type-I collagen in the chondral layer was maintained at a level higher than eight in the whole culture range without significant differences (Figure 7B), meaning that the collagen amount in matrix for the chondral layer overwhelmingly belongs to type-II collagen. These results provide evidence for the proliferation and phenotype preservation of chondrocytes in the chondral layer.

Similarly, type-I collagen amount in the bony layer of the layered scaffolds significantly increased as the culture-time advanced (Figure 7C), proving that osteoblasts are able to persistently synthesize bone-related matrix. Alkaline phosphatase (ALP) was used as a marker to check the osteoconductive potential of the bony layer in scaffolds since it is a key regulatory enzyme in the mineralization process of neotissue [37]. As shown in Figure 7C, ALP activity increased rapidly from day 1 to day 7, reached a high level after 14 days, and thereafter, was maintained at the same level (*p* > 0.05). High ALP activity at day 7 can be considered as the onset of mineralization, and the highest ALP activity level at day 14 should be related to the maturation of the osteocytes [37,38]. Results in Figure 7C suggest that the composition and structure of the bony layer support the mineralization of neotissue, and this layer in the layered scaffold is capable of mimicking the functions of subchondral bone layer in the osteochondral ECM. 

On the basis of above-presented results, it can be reached that the presently developed nanofibrous membrane unit can play three key roles in tightly connecting the chondral layer and the bony layer in the layered scaffolds; allowing for certain molecules with limited sizes to pass through, but preventing the cells that are seeded in the chondral layer and the bony layer from migrating cross, which makes the nanofibrous membrane unit like the calcified layer in the osteochondral ECM. Further studies on the mechanical properties and degradation tolerance of the layered scaffolds as well as their in vivo performance for osteochondral repair are now underway, and the relevant results will be presented in separate reports.

## 3. Materials and Methods

### 3.1. Materials

Chitosan (CH) powder and poly (ethylene oxide) (PEO, *M*_w_: ca.6 × 10^5^) were purchased from Aladdin, China. Deacetylation degree and viscosity-average molecular weight of CH were measured as 92.6 (±1.9)% and 4.1 (±0.16) × 10^6^, according to reported methods [39]. Glucose and fluorescein isothiocyanate labeled dextran (FITC-DEX) were purchased from Sigma-Aldrich (St. Louis, MO, USA). All other chemicals were of analytical grade and bought from Sinopharm, China.

### 3.2. Preparation of Silk Fibroin

Silk fibroin (SF) was isolated from *Bombyx mori* cocoons (Hubei Academy of Agriculture Sciences, China) following methods described elsewhere [40]. In brief, cocoons were degummed twice in a 0.5% (*w*/*v*) NaHCO_3_ solution at 100 °C for 40 min. After being washed with distilled water, the product was dissolved in a ternary solvent (CaCl_2_/CH_3_CH_2_OH/H_2_O, ca.1/2/8 molar ratio) stirred at 80 °C for 1 h. After centrifugation at 7000 rpm for around 10 min, the resulting mixture was dialyzed against distilled water for 3 days using membrane tubes (MW cutoff: 3500) to remove impurities. The resulting SF solution was lyophilized for further use.

### 3.3. Synthesis of Hydroxyapatite Nanoparticles

Hydroxyapatite (HA) nanoparticles (NPs) were prepared while using a co-precipitation method [40]. The content of calcium and phosphorus in HA NPs (spindle-like shape, average length: 107.6 ± 13.2 nm) was measured by titrating ethylene diamine tetraacetic acid complexes for the former [41], and precipitating phosphomolybdate quinoline complexes for the latter [42]. The stoichiometric Ca/P ratio in HA NPs was determined as about 1.67.

### 3.4. Preparation of Nanofibrous Membranes

Electrospinning dopes with various compositions were formulated and their major parameters are listed in Table 1. Briefly, given amounts of CH (200, 250, 300, and 350 mg) were, respectively, dissolved in 2mL of 90% aqueous acetic acid to prepare four solutions having different concentrations. To each solution, prescribed amounts of HA and PEO were slowly introduced with stirring for 24 h to prepare different CH/HA/PEO mixtures. To each mixture, concentration-varied SF solutions in distilled water were added to prepare four kinds of CH/SF/HA/PEO dopes and the final volume of each dope was 5 mL. After additional 12 h stirring, dopes were subjected to electrospinning. The electrospinning system was run under the following conditions: voltage, 25 kV; feeding rate, 1.5 mL/h; temperature, 25 °C; and, tip-collector distance, 12–18 cm. Four kinds of nanofibrious membranes with similar thickness of around 150 μm but with different compositions were spun under the same conditions and their thickness was controlled by changing the electrospinning time, as indicated in Table 1.

### 3.5. Preparation of Layered Scaffolds

A type of nanofibrous membrane unit was first fabricated using formulations that are shown in Table 1, and following the same processing conditions mentioned above. In brief, a nanofibrous membrane similar to NFM(4) was electrospun, and it was served as the bottom layer. Atop this bottom layer, a new layer similar to NFM(3) was electrospun. Similarly, this process was repeated until the top layer that was similar to NFM(1) was electrospun. An integral nanofibrous membrane unit was formed during the consecutive spinning procedures. CH, SF, and HA in this membrane unit had their respective gradients changing in their own way, as shown in Table 1. The obtained nanofibrous membrane unit was fully neutralized in a 4% NaOH solution for 8 h and repeatedly washed with deionized water until neutrality was achieved, followed by lyophilization. This type of nanofibrous membrane unit was used as mimetic calcified layer for subsequent fabrication of layered scaffolds.

The layered scaffolds were built using a method somewhat similar to that as described elsewhere [43]. The bottom layer (bony layer) of the scaffold was first constructed using a 2 wt% CH/HA composite solution (weight ratio of CH to HA: 52/48) in 1% aqueous acetic acid. The prebuilt nanofibrous membrane unit was superimposed over this bottom layer to serve as a new layer to function as the mimetic calcified layer. Similarly, onto the surface of the mimetic calcified layer, a top layer (chondral layer) was built while using a 3 wt% CH/SF composite solution (weight ratio of CH to SF: 32/68) in 1% aqueous acetic acid. After being freeze-dried, the scaffolds were neutralized in a 1 wt% NaOH solution, washed with distilled water to reach neutrality, and followed by lyophilization. The layered scaffolds were further crosslinked using a 10% sodium tripolyphosphate solution at room temperature for 4h, repeatedly washed with deionized water, and lyophilized again.

### 3.6. Characterization

HA NPs were viewed using a transmission electron microscope (TEM, 3H-7000FA, Hitachi, Tokyo, Japan). X-ray diffraction (XRD) patterns of HA NPs were recorded on an X-ray diffractrometer (X’Pert PRO, Philips, Netherlands). HA powder was filled into aluminum frames (30 mm × 30 mm) and tightly compacted. HA NP samples were scanned from 5 to 80° (2*θ*) at a rate of 2°/min. Nanofibrous membranes (8 × 8 mm^2^) and layered scaffolds (section size: ca. 8 × 4 × 4 mm^3^) were viewed with scanning electron microscope (SEM, Quanta 200, FEI, Hillsboro, OR, USA or Sirion 200, FEI, Hillsboro, OR, USA) after gold-spraying. In cases of cross-section observation of nanofibrous membrane units or layered scaffolds, the units or the scaffolds were cut along the direction perpendicular to the surface of layers in the units or in the scaffolds. The average diameter of nanofibers was determined by averaging 200 different fibers in SEM images with the aid of analysis software (ImageJ). Scaffolds were horizontally sectioned into sheets (discs: 8 mm in diameter; 0.8–1.6 mm in thickness) approximately along interfaces between different layers by means of a Teflon mold with a movable stainless steel mandrel [43]. Porosity and mean pore size of the sheets were determined using a mercury intrusion porosimeter (AutoPore IV 9500, Micromeritics, Norcross, GA, USA). Mercury was filled progressively, changing from a pressure of 3 kPa to one of 300 MPa. The relationship between the pressure and the minimum size pore is [44]:

*d* = −4γcos*θ*/*P* (1)

where γ is the surface tension, *θ* is the contact angle between mercury and the sample, and *P* is the pressure that is required to force mercury into a pore with a diameter of *d*. All of these measurements were performed at least three times.

### 3.7. Permeability Evaluation

Cylindrical dry scaffolds (10 mm in diameter) were horizontally cut into sheets approximately along interfaces between different layers. Diffusive permeability of sheets was measured using glucose (*M*_w_ 180 Da) and FITC-DEX (*M*_w_, 4 kDa, 10 kDa, and 70 kDa) as model molecules. The solutes were respectively dissolved in PBS to prepare 4 kinds of solutions with the same concentration of 1.0 mg/mL. In a typical measurement procedure, a sheet was sandwiched between two stainless steel meshes to form into a fixture. The fixture was mounted on one end of a 2 mL glass tube (inner diameter: 10 mm) and the rim of the fixture was sealed with a cyanoacrylate sealant. One of solute-contained solutions (0.5 mL) was introduced into the glass tube, and the sheet-mounted end of the tube (diffusion area of sheet: ca. 0.6 cm^2^) was immersed in 4 mL of PBS (pH 7.4) in a 10 mL vial to a proper depth so that the liquid level inside the tube was equal to the level in the vial. The vial was maintained at 37 °C with stirring at 60 rpm. At predetermined time points, 1.5 mL of medium was retrieved from the vial with replenishing the same volume of fresh PBS and the glucose or FITC-DEX amount diffused across the sheet was analyzed by Glucose Essay Kit (Sigma) and UV-vis spectrometry, respectively.

### 3.8. Cell Culture

Animal experiments were conducted according to NIH standards, as set forth in the guide for the care and use of laboratory animals. 

Articular cartilage was aseptically harvested from the knee joints of New Zealand white rabbits (four-week old). Cartilage samples were diced into pieces. The extracellular matrix of samples was predigested with a 0.2% trypsin solution for 2 h and subsequently digested with a 0.2% collagenase II solution at 37 °C for 10 h or more. The retrieved chondrocytes were expanded in DMEM that was supplemented with 10% fetal calf serum (FCS), 100 U/mL penicillin, and 100 μg/mL streptomycin, and the culture medium was replaced every two days. 

Since osteoblasts are difficult to be isolated from the bone tissues of adult animals, neonatal New Zealand white rabbits (24-h old) were thus employed for osteoblast isolation. Cervical bone was first aseptically isolated from the rabbits, and the bone samples were then minced into pieces after removal of the adherent tissue, followed by digestion in 0.25% trypsin solution at 37 °C for 15 min while being shaken on an orbital shaker. The collected bone pieces were then cultured in the culture flask containing 20% FCS and antibiotics in a humid 5% CO_2_ atmosphere with culture medium change twice a week. The isolated osteoblasts were expanded using DMEM, supplemented with 10% FCS, penicillin (100 U/mL), and streptomycin (100 μg/mL) with medium refreshment every three days.

Cylindrical layered scaffolds (5 mm in diameter, ca.4.0 mm in thickness) were sterilized with ethylene oxide gas and were used for the cell culture. Details for seeding cells and culturing the cell-seeded scaffold in the two-compartment chamber were schematically illustrated in Figure S2. In a typical procedure, the scaffold was mounted to the hole on the separation plate in such a way that the mimetic calcified layer inside the layered scaffold was just fitted the hole of the separation plate, whereas the chondral layer and the bony layer of the scaffold were protruded from both sides of the separation plate, respectively. The scaffold edge that was in contact with the hole of the separation plate was sealed with waterproof tape that was sterilized using epoxyethane gas prior to use. The mounted scaffold was pre-wetted over night, chondrocytes (2 × 10^6^ cells) were seeded onto the chondral layer of the scaffold and cultured in complete culture medium on standard conditions for 2 h. The scaffold-mounted separation plate was then inverted, and osteoblasts (10^6^ cells) were seeded to the bony layer of the scaffolds, followed by another 2 h culture. After that, the separation plate was inserted into the two-compartment chamber with the addition of pre-warmed complete culture medium into two compartments, respectively. Cell culture was carried out for various durations up to 21 days with medium replacement every two days, and cells that were grown in tissue culture plate were used as control.

At the end of preset culture intervals, cell-seeded scaffolds were vertically sectioned into slices. Viability of cells was evaluated using a calcein-AM/ethidium homodimer-1(EthD-1) Live/Dead Kit (Molecular Probes). After staining, cells were visualized in a downward manner at an image plane depth of about 100 μm using confocal scanning microscope.

To assess cell proliferation in both chondral layer and bony layer of the layered scaffold, the scaffolds containing cells were transversely sectioned and the resulting sheets matching with the chondral layer and the bony layer were subject to measurements using a MTT assay, respectively. The optical density for determining cell numbers was measured at 570 nm using a microplate reader against a blank sodium dodecyl sulfate solution. The sheets without cell seeding were used as negative control. All the above mentioned measurements were performed at least three times.

### 3.9. Matrix Deposition Analysis

The sheets cut from the chondral layer and the bony layer of the fixed and dehydrated cell-seeded scaffolds were sectioned into slices, and the slices were stained with alcian blue (1%) for GAG deposition in the chondral layer or alizarin red (2%) for calcium deposition in the bony layer. Type-II collagen in the chondral layer and type-I collagen in the bony layer were evaluated using immunostaining. Briefly, deparaffinized and rehydrated slices were incubated with 0.1% Triton X-100 in PBS for 15 min and blocked with 5% bovine serum albumin in PBS for 30 min. They were then incubated with the primary antibodies (Rabbit polyclonal to collagen II for chondrocytes, Rabbit polyclonal to collagen I for osteoblasts, Abcam). Slices were washed and incubated with an Alexa Fluor 488 labeled secondary antibody (Abcam) for type-II collagen or an Alexa Fluor 647-labeled secondary antibody (Abcam) for type-I collagen.

GAG and type-II collagen contents in the chondral layer were also quantified. Briefly, sheets that were matching with the chondral layer were minced into pieces and were homogenized in PBS using a glass grinder. The resulting pieces were exposed to 0.5% Triton X-100 in PBS, followed by three frozen-thaw cycles. The homogenates were centrifuged (15 min, 5000 rpm) to achieve supernatants. GAG amount in supernatants was determined using a modified 1,9-dimethylmethylene blue (DMMB) binding assay with chondrotin-6-sulfate as the standard [44]. Type-II collagen amount was measured using a collagen type II ELISA kit (rabbit) (US Biological, Salem, MA, USA). Similarly, type-I collagen content was also quantified using a collagen type I ELISA kit (rabbit) (US Biological, Salem, MA, USA). Alkaline phosphatase (ALP) activity was assessed while using an ALP assay kit (Abcam, Cambridge, MA, USA), and the ALP activity was normalized to the total protein content [45]. These quantitative measurements were performed following the protocols and instructions provided by manufactures. All of these measurements were performed in triplicate. 

### 3.10. Statistical Analysis

Data were presented as mean ± standard deviation. Two-way analysis of variance was conducted for statistical analysis between groups, and the statistical difference was considered to be significant when the *p* value was less than 0.05.

## 4. Conclusions

Nanofibrious membrane unit with compositional and structural gradients was successfully fabricated by using chitosan, silk fibroin and hydroxyapatite composites and employing an electrospinning method. By integrating this specially designed nanofibrious membrane unit into the three-layer scaffolds, the unit was able to act as a well-devised connection layer to tightly bind the chondral layer and the bony layer together, allow for some molecules with limited molecular weight to permeate through, and effectively prevent the seeded cells from migrating cross, confirming that the nanofibrious membrane unit can function very like a calcified layer in the native osteochondral ECM. The optimally assembled scaffolds were capable of supporting the growth of different cells, respectively, seeded in their chondral layer and bony layer, and promoting the matrix deposition with cell-specified characteristics. Matrix analyses supported the phenotype preservation of chondrocytes in the chondral layer and the mineralization of neotissue in the bony phase. Results confirm that this new type of layered scaffolds can partially mimic the chondral layer, calcified layer, and subchondral bone layer in osteochondral matrix, suggesting that they have potential for applications in osteochondral repair.

## Figures and Tables

**Table 1:** Parameters of nanofibrious membranes.

Sample Name	Chitosan (wt%)	SF (wt%)	HA (wt%)	PEO (wt%)	Spinning Time (min)	Average Thickness (μm)	Average Diameter of Fibers (nm)	Average Pore Size (μm) ^(b)^	Average Porosity (%) ^(c)^
NFM(1) ^(a)^	36	50	10	4	265	157.6 ± 6.84	212.6 ± 23.54	2.8 ± 0.53	13.2 ± 1.64
NFM(2)	40	39	17	4	220	148.2 ± 8.13	231.2 ± 13.39	4.53 ± 0.92	18.4 ± 2.16
NFM(3)	44	28	24	4	175	153.1 ± 7.09	250.8 ± 11.71	6.62 ± 1.03	24.5 ± 2.71
NFM(4)	48	17	31	4	150	159.9 ± 9.61	267.4 ± 10.83	9.05 ± 1.14	31.7 ± 3.09

^(a)^ NFM(*i*) (*i* = 1, 2, 3, 4) membranes were prepared by changing tip-collector distance from12 to 18 cm with a step-size of 2 cm while maintaining all other procesisng conditions constant. ^(b)^ Significant differences (*p* < 0.05) in average pore-sizes were registered among NFM(*i*) (*i* = 1, 2, 3, 4) membranes. ^(c)^ Significant differences (*p* < 0.05) in average porosities were registered among NFM(*i*) (*i* = 1, 2, 3, 4) membranes.

## Supplementary Materials

Supplementary materials can be found at http://www.mdpi.com/1422-0067/19/8/2330/s1.
