# Supplementary material for: Chitosan-Based Nanofibrous Membrane Unit with Gradient Compositional and Structural Features for Mimicking Calcified Layer in Osteochondral Matrix
Source: Int J Mol Sci. 2018 Aug 8;19(8):2330. doi: 10.3390/ijms19082330 (PMC6121876; doi:10.3390/ijms19082330)
Supplement: Supplementary file 1 [file ijms-19-02330-s001.pdf]

Supporting information

Layered scaffolds incorporated with compositional gradient nanofibrous membranes for mimicking the calcified layer in osteochondral matrix

Jiaoyan Liu[1], Qing fang[1], Xiaofeng Yu[1], Ying Wan[1,*], Bo Xiao[2,*]

[1] College of Life Science and Technology, Huazhong University of Science and Technology, Wuhan 430074, P. R. China

[2] Institute for Clean Energy and Advanced Materials, Faculty for Materials and Energy, Southwest University, Chongqing 400715, P. R. China

*Corresponding authors: Dr. Ying Wan, E-mail: ying_wan@hust.edu.cn (Y. Wan); Tel: 86-27-87792147;Fax: 86-27-87792234; Dr. Bo Xiao,Tel: 86-23-68254762

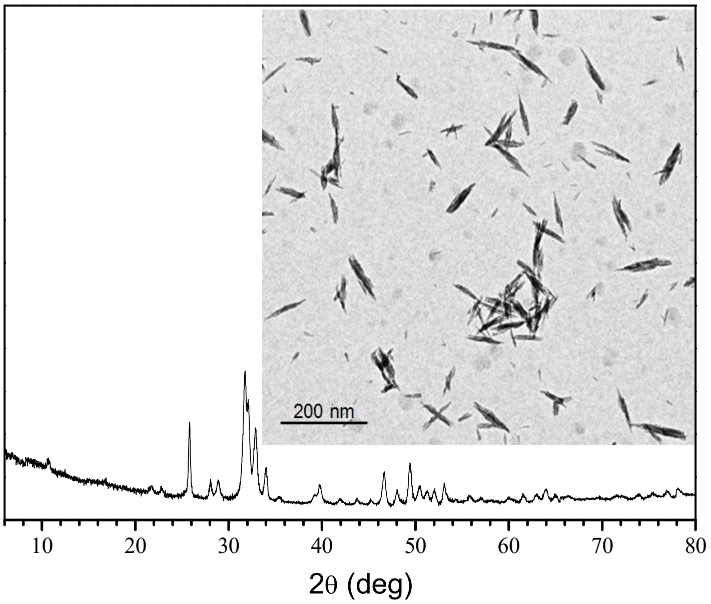

Figure S1. Typical XRD pattern and a representative TEM image for hydroxyapatite nanoparticles.

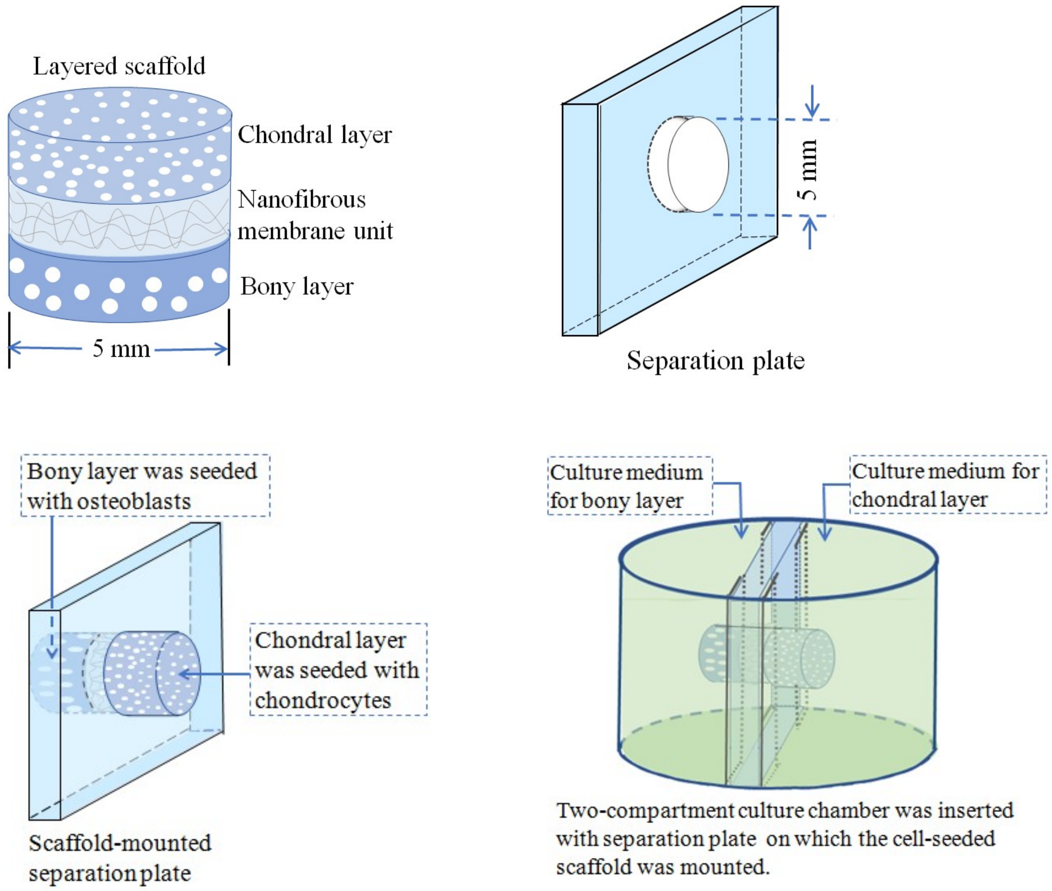

Figure S2. Schematic illustration for seeding cells and the followed culture of.cell-seeded scaffolds in the two-compartment chamber.

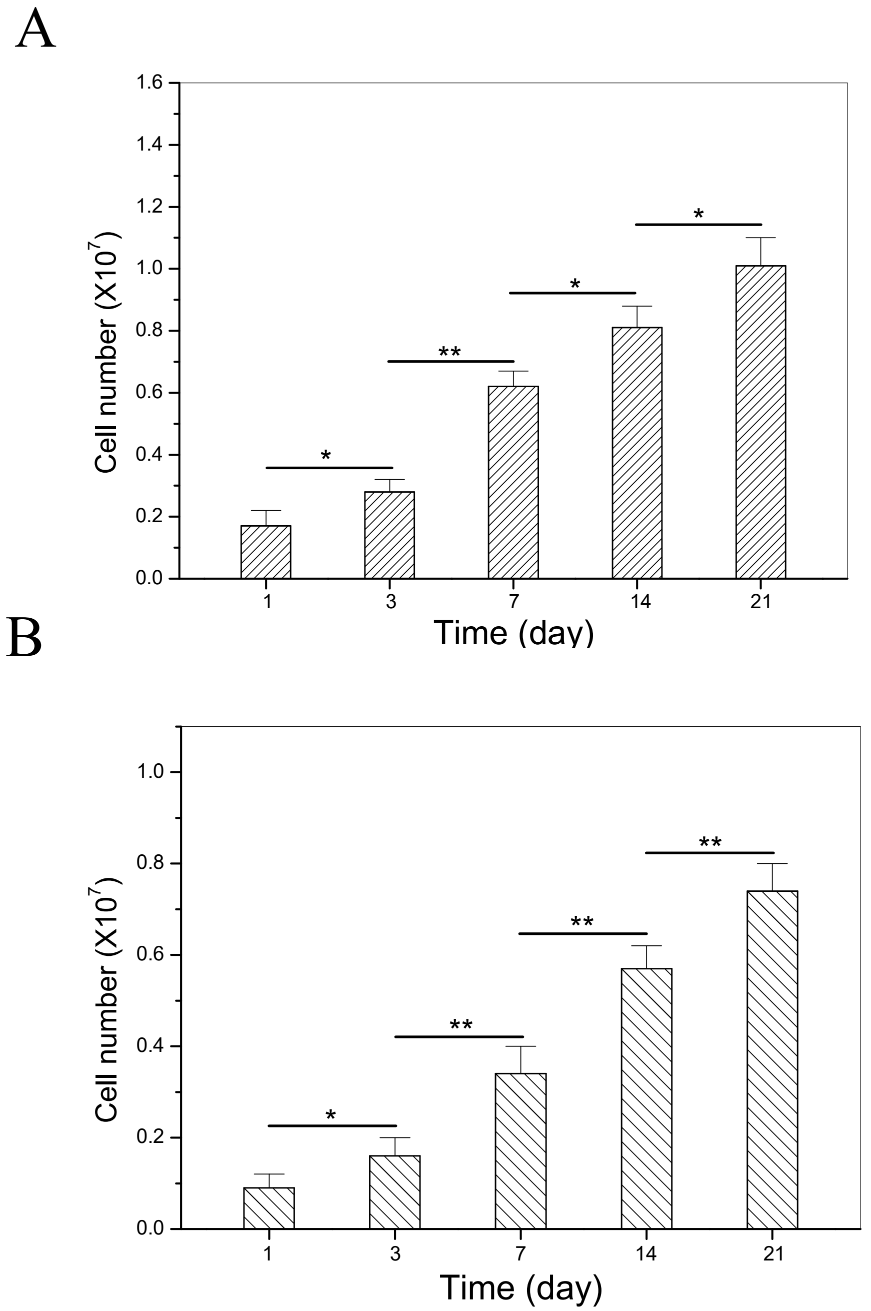

Figure S3. Chondrocyte proliferation (A) in chondral layer and osteoblast; proliferation (B) in bony layer of the layered scaffolds (*, $p<0.05$; **, $p<0.01$) .
